# Geographical distribution and spatial autocorrelation of newborns with orofacial clefts: an epidemiological study, Paraná, 2018-2022

**DOI:** 10.1590/S2237-96222025v34e20250189.en

**Published:** 2025-10-31

**Authors:** Mariana Martire Mori, Camila Moraes Garollo Piran, Alana Vitória Escritori Cargnin, Márcia Moroskoski, Carlos Alexandre Molena Fernandes, Débora Regina de Oliveira Moura, Marcela Demitto Furtado

**Affiliations:** 1 Universidade Estadual de Maringá, Maringá, PR, Brazil

**Keywords:** Spatial Analysis, Infant, Newborn, Cleft Lip, Cleft Palate, Epidemiologic Studies, Análisis Espacial, Recién Nacido, Labio Leporino, Fisura del Paladar, Estudios Epidemiológicos

## Abstract

**Objective:**

To examine the geographical distribution and spatial autocorrelation of cases of orofacial clefts in newborns in Paraná.

**Methods:**

This study employed epidemiological methods using data from the Live Birth Information System for the years 2018-2022. The rates were obtained by dividing the number of live births with orofacial clefts by the total number of births in each year and city, and then multiplying by 1,000. The Global Moran’s I was calculated, and spatial autocorrelation was assessed.

**Results:**

During the study period, 574 cases of newborns with orofacial clefts were analyzed, distributed across 163 cities in Paraná. The rates ranged from 0.00 to 2.44 cases per 1,000 live births. High-high clusters were present in Londrina, Cornélio Procópio/Bandeirantes, Ivaiporã, Cascavel, Foz do Iguaçu, Francisco Beltrão, Dois Vizinhos, Laranjeiras do Sul/Quedas do Iguaçu, Pato Branco, Guarapuava, and Paranaguá, with rates ranging from 1.10 to 2.44 cases per 1,000 live births.

**Conclusion:**

The study highlighted the spatial heterogeneity of newborn cases with orofacial clefts in Paraná, underscoring the need to investigate the causes of orofacial clefts, which will contribute to preventing new cases and enhancing public health actions.

## Resumen

**Objetivo:**

Examinar la distribución geográfica y la autocorrelación espacial de los casos de fisura orofacial en recién nacidos en Paraná.

**Métodos:**

Se trató de un estudio epidemiológico que utilizó datos del Sistema de Información sobre Nacidos Vivos, correspondientes al período 2018-2022. Las tasas se obtuvieron dividiendo el número de nacidos vivos con fisura orofacial por el total de nacimientos en cada año y municipio, multiplicado por 1.000. Se calculó el índice global de Moran y se evaluó la autocorrelación espacial.

**Resultados:**

Se analizaron 574 casos de recién nacidos con fisura orofacial en el período estudiado, distribuidos en 163 municipios de Paraná. Las tasas variaron de 0,00 a 2,44 casos por 1.000 nacidos vivos. Se identificaron agrupamientos tipo alto-alto en Londrina, Cornélio Procópio/Bandeirantes, Ivaiporã, Cascavel, Foz do Iguaçu, Francisco Beltrão, Dois Vizinhos, Laranjeiras do Sul/Quedas do Iguaçu, Pato Branco, Guarapuava y Paranaguá, con tasas que variaron de 1,10 a 2,44 casos por 1.000 nacidos vivos.

**Conclusión:**

El estudio evidenció la heterogeneidad espacial de los casos de recién nacidos con fisura orofacial en Paraná, haciendo imprescindible la investigación de las causas de la fisura orofacial, lo cual contribuye a la prevención de nuevos casos y al fortalecimiento de las acciones de salud pública.

**Ethical aspects**

This research respected ethical principles, having obtained the following approval data:

Research Ethics Committee: Universidade Estadual de Maringá

Opinion number: 7.041.250

Approval date: 29/8/2024

Certificate of Submission for Ethical Appraisal: 81990024.0.0000.0104

Informed Consent Form: Exempt.

## Introduction 

Orofacial clefts are among the most common craniofacial anomalies, and their incidence is increasing. In 2020, the national prevalence of orofacial clefts was 5.25 per 10,000 live births. In the country, between 1999 and 2020, the South and Southeast regions had the highest rates, at 6.34 and 4.26 per 10,000 live births (1). Between 2008 and 2017, the incidence of orofacial clefts in southern Brazil was 74.6 per 100,000 live births, followed by Rio Grande do Sul with 71.8 per 100,000 live births, and Paraná with 68.4 per 100,000 live births (2).

Cases vary according to racial, geographical, and socioeconomic factors (3). Between 1999 and 2020 in Brazil, there was a predominance of males with cleft lip and palate of the White race/skin color (1). In South Korea, among children with cleft lip and/or palate born between 2006 and 2018, it was found that children with orofacial clefts had a higher risk of premature birth and neonatal death when compared to newborns without the anomaly (3).

This malformation can lead to complications in breastfeeding and feeding, speech alterations, psychosocial challenges, problems with self-image, and difficulties with resilience. Many of these problems can impact the family system as a whole, whether through family disruptions, marital crises that culminate in divorce, estrangement from other children, or job abandonment (4-6).

Despite the relevance of the topic, few national studies are exploring the spatial distribution of orofacial clefts. International studies analyzing the spatial distribution of malformations, carried out in Cuba, Iran, China, and South Korea, have identified areas with the highest number of cases of orofacial clefts, helping to elucidate the etiology due to environmental factors (7-10). In Brazil, however, this approach is still little explored, limiting our understanding of the distribution of cases.

This study proved necessary to contribute to the epidemiological mapping of clefts and aimed to examine the geographical distribution and spatial autocorrelation of cases of orofacial clefts in newborns in Paraná.

## Methods 

### Design 

This study examined the epidemiology of orofacial cleft rates in live births in Paraná between 2018 and 2022, utilizing the Live Birth Information System. The study complied with the Strengthening the Reporting of Observational Studies in Epidemiology (STROBE) guidelines (11).

### Setting 

Located in the southern region of Brazil, Paraná covers an area of 199,298 km^2^, and in 2022, its estimated population was 11,444,380 inhabitants. The state is divided geographically into 10 mesoregions and 399 cities, with a Human Development Index (HDI) of 0.769, indicating a high index rate (12,13). The state is subdivided into 29 immediate regions, which correspond to groups of cities that share an urban center as a reference point for their needs, primarily in areas such as health, education, labor, shopping, and consumption (14).

### Data source and measurement

The data were collected in June 2024, using the Live Birth Information System and population estimates, which are available at the Information and Informatics Department of the Brazilian National Health System (15,16).

To map the spatial distribution of live births with orofacial clefts, rates were calculated for each of Paraná’s cities, considering the analysis to be carried out. The rates were calculated using the ratio between live births with orofacial clefts and the population of live births in each year and location, multiplied by 1,000.

### Statistical methods

For the spatial analysis, the criterion of spatial neighborhood and Queen-type contiguity between the cities of Paraná was considered. The global Moran’s I was calculated, and the degree of spatial autocorrelation of the birth of newborns with orofacial clefts between cities was assessed. 

The local Moran’s I was applied to identify spatial clusters, and these were classified into three types: high-high - high rates in cities surrounded by similarly high areas; low-low - low rates in cities surrounded by areas with low rates; and low-high - low rates in cities that border areas with high rates (17).

QGIS software was used for spatial distribution analysis, and GeoDa was used to identify spatial clusters. R software, version 4.0.2 (R Core Team, 2020), and R-INLA, version 20.03.17, were used for exploratory data analysis and modeling. To calculate the spatial distribution, the cartographic base of Paraná, which contains the municipal boundaries and is available online in geographic vector format, was used (18).

## Results 

**Figure 1:**
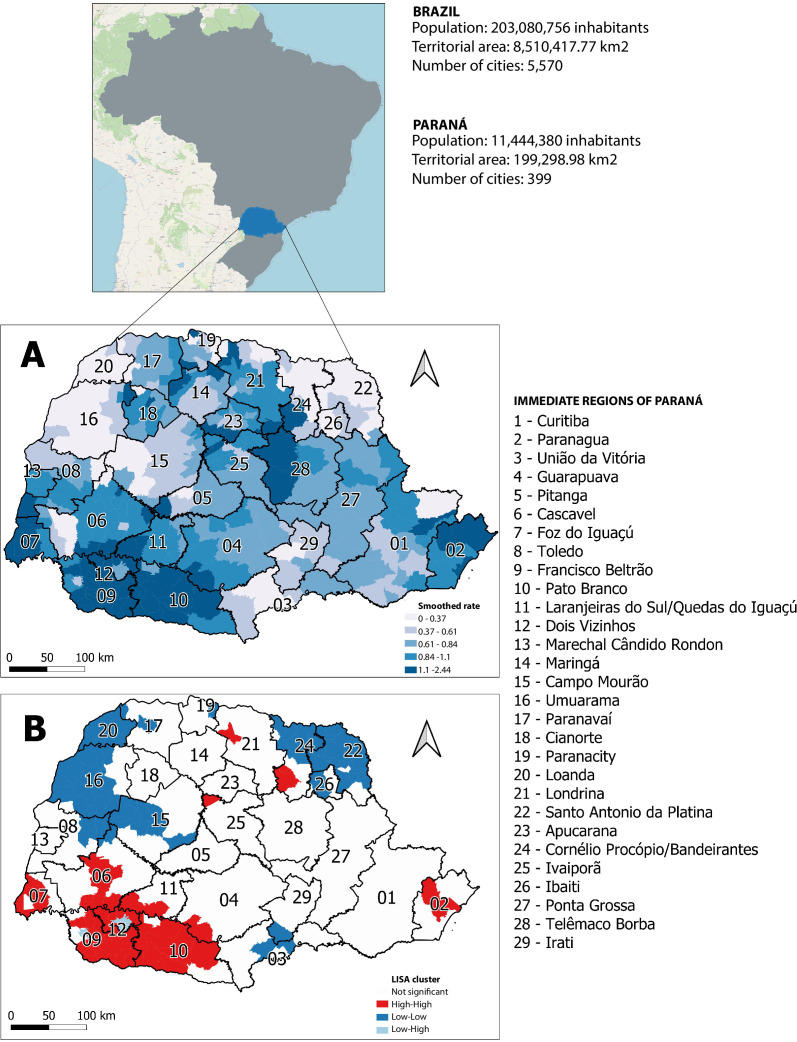
Spatial distribution of smoothed rates of orofacial clefts per 1,000 live births (A) and their clusters (B). Paraná, 2018-2022

Between 2018 and 2022, 574 cases of newborns with orofacial clefts in Paraná were analyzed, distributed across 163 municipalities in the state. The Global Moran’s I indicated strong positive autocorrelation (p-value 0.001). The spatial distribution of the smoothed rates is shown in Figure 1A, where the smallest gradients represented the lowest rates and the most pigmented gradients showed the highest rates, ranging from 0.00 to 2.44 cases per 1,000 live births. The immediate regions with high rates (between 2.44) were mainly located in the southwest and north of the state, including Paranaguá (a port city), Pato Branco, Francisco Beltrão, Dois Vizinhos, Foz do Iguaçu, Telêmaco Borba, and Cornélio Procópio/Bandeirantes. 

The immediate regions with the lowest smoothed rates of newborns with orofacial clefts were located in the northeast and northwest of Paraná, namely Loanda, Umuarama, Campo Mourão, Santo Antônio da Platina, Cornélio Procópio/Bandeirantes, Ibaiti, Irati, and União da Vitória, ranging from 0.00 to 0.37 cases per 1,000 live births.

The results of local spatial autocorrelation indicated a pattern of cities with high rates surrounded by cities with high rates (high-high), significant in the southwest of the state, and cities with low rates surrounded by cities with low rates (low-low), mainly in the northeast and northwest of the state (Figure 1B).

The cases of orofacial cleft showed high-high clusters in the immediate regions of Londrina, Cornélio Procópio/Bandeirantes, Ivaiporã, Cascavel, Foz do Iguaçu, Francisco Beltrão, Dois Vizinhos, Laranjeiras do Sul/Quedas do Iguaçu, Pato Branco, Guarapuava, and Paranaguá.

The immediate regions of Loanda, Paranavaí, Paranacity, Cornélio Procópio/Bandeirantes, Santo Antônio da Platina, Ibaiti, Umuarama, Toledo, Cascavel, Campo Mourão, União da Vitória, and Irati had low-low clusters.

## Discussion 

The spatial pattern of orofacial cleft cases in Paraná revealed a non-random distribution, with clusters exhibiting high rates primarily in the southwest and north of the state. This pattern indicates that there may be common factors between the regions that influence the occurrence of the malformation.

As this is an epidemiological study of spatial distribution, there were some limitations in interpreting the results. It was not possible to establish a causal relationship or identify the etiological factors for the occurrence of orofacial clefts. The lack of detailed information made it impossible to measure the known risk factors for orofacial clefts in each region.

Despite these limitations, the identified patterns suggest the presence of structural inequalities between the cities. The disparity in cases of orofacial clefts between the cities of Paraná may be related to the economic development of the regions and access to health care. It was evidenced that most cases of newborns with orofacial clefts occurred in mothers with lower educational level, lower income, and inadequate access to prenatal consultations, which indicates the importance of the social context for child health (19). 

The relevance of folic acid consumption before and during pregnancy to avoid cases of orofacial clefts was highlighted (20,21). A meta-analysis conducted by researchers from Canada confirmed, in their findings, that the use of folic acid is a protective factor for orofacial clefts, especially when used before conception (20). In Ethiopia, between 2016 and 2018, 64.5% of mothers who did not use folic acid had children with orofacial clefts (21). 

Adherence to supplementation may be related to the quality of prenatal care and the knowledge of pregnant women (21). The characteristics of prenatal care were evaluated concerning the guidelines offered to pregnant women in a maternity hospital in Londrina. In this context, it was identified that women received an ideal number of visits; however, the quality of care ranged from intermediate to inadequate, with poor access to information in prenatal care (22). It was identified that the Londrina region presented high-high type clusters for cases of orofacial clefts, which could be related to these factors.

Confounding factors such as multiparity, inadequate diet with nutrients at levels lower than recommended, and advanced maternal age should also be considered in the investigation of cases of orofacial clefts, which highlights the complexity of the malformation (23).

In southern Iran, urban areas had a higher incidence of orofacial clefts than rural areas, suggesting that the risk of maternal exposure to high levels of air pollutants in large cities is associated with the degree of urbanization and the emission of industrial pollutants (8). This fact can be applied to the urban centers of Paraná, such as Londrina, a center recognized for its commerce and industry, which has presented high rates of orofacial clefts. 

The causes of orofacial clefts, as well as other congenital malformations, are not completely explained. However, the importance of the interaction between genetic and environmental aspects is noted. Among the environmental factors, exposure to pesticides considerably increases the chances of developing orofacial cleft, associated or not with other anomalies (24,25). Pesticides are widely used in Paraná, as the state is the second-largest grain producer (26) and holds the second national position in pesticide use. Between 2013 and 2020, pesticide consumption in the state increased by 14.55%, rising from 8.87 kg/ha to 9.82 kg/ha. The central-southern, western, and central-eastern regions showed values above the state average. This intensive use may be related to the higher incidence of orofacial clefts in regions such as Cascavel and Londrina, where consumption per hectare is high (27).

Among lactating residents of Francisco Beltrão, the presence of glyphosate (a broad-spectrum herbicide widely used in the southwest of Paraná) was found in all samples collected from breast milk, as well as in water, revealing that the population was completely exposed to pesticides. The data corroborate the finding of this study, as the index of orofacial cleft in newborns from the immediate region of Francisco Beltrão was among the highest (28).

The low-low clusters in cities located mainly in the northeast and northwest of Paraná may not necessarily reflect lower risk. However, they may be related to underreporting of cases of orofacial cleft in newborns due to the turnover of health professionals, lack of training, or low investment in screening, diagnosis, and access to treatment of children with this congenital anomaly (19,29).

This study contributed to partially reveal the panorama of cases of orofacial cleft in Paraná and was innovative in using a method of analysis that is little adopted nationally for this malformation. The findings identified risk sites for newborns with orofacial clefts, which support health managers in reflecting on access to prenatal care, as well as on risk indicators at the area level, which should be considered a priority when developing measures to reduce the occurrence of cleft lip and/or palate (9).

The research highlighted the spatial heterogeneity of birth rates with orofacial clefts in Paraná. Municipal clusters with high rates of orofacial clefts in newborns were identified, indicating possible regional inequalities related to social and environmental factors and access to health care. Although the study did not make causal inferences, the findings suggested that regions with higher incidence may reflect both higher risk exposure and better detection, while areas with low incidence may indicate underreporting. 

It is essential to investigate the variables that may be influencing orofacial cleft rates in Paraná, in order to strengthen public health actions and prevent an increase in cases. Additionally, environmental factors, such as exposure to pesticides and pollutants, which may contribute to an increased incidence in urban and agricultural regions, should be investigated in more detail. 

It is recommended that further studies on the topic be undertaken, employing varied methodological approaches and a distinct analytical framework. These studies should provide deeper support that corroborates the development of preventive strategies and the adaptation of public policies to regional realities.
