# Supplementary material for: Geographical distribution and spatial autocorrelation of newborns with orofacial clefts: an epidemiological study, Paraná, 2018-2022
Source: Epidemiol Serv Saude. 2025 Oct 31;34:e20250189. doi: 10.1590/S2237-96222025v34e20250189.en (PMC12582527; doi:10.1590/S2237-96222025v34e20250189.en)
Supplement: Translation

# Translation: Distribuição geográfica e autocorrelação espacial de recém-nascidos com fissura orofacial: estudo epidemiológico, Paraná, 2018-2022

**DOI:** 10.1590/S2237-96222025v34e20250189.pt

**Authors:** Mariana Martire Mori, Camila Moraes Garollo Piran, Alana Vitória Escritori Cargnin, Márcia Moroskoski, Carlos Alexandre Molena Fernandes, Débora Regina de Oliveira Moura, Marcela Demitto Furtado

**Affiliations:** 1 Universidade Estadual de Maringá, Maringá, PR, Brasil

**Keywords:** Análise Espacial, Recém-Nascido, Fenda Labial, Fissura Palatina, Estudos Epidemiológicos

## Abstract

**Objetivo:**

Examinar a distribuição geográfica e a autocorrelação espacial dos casos de fissura orofacial em recém-nascidos no Paraná.

**Métodos:**

Tratou-se de estudo epidemiológico que utilizou dados do Sistema de Informações sobre Nascidos Vivos, referentes ao período 2018-2022. As taxas foram obtidas dividindo o número de nascidos vivos com fissura orofacial pelo total de nascimentos em cada ano e município, multiplicado por 1 mil. O índice de Moran global foi calculado e avaliou-se a autocorrelação espacial.

**Resultados:**

Foram analisados 574 casos de recém-nascidos com fissura orofacial no período estudado, distribuídos em 163 municípios do Paraná. As taxas variaram de 0,00 a 2,44 casos por 1 mil nascidos vivos. Os agrupamentos do tipo alto-alto estiveram presentes em Londrina, Cornélio Procópio/Bandeirantes, Ivaiporã, Cascavel, Foz do Iguaçu, Francisco Beltrão, Dois Vizinhos, Laranjeiras do Sul/Quedas do Iguaçu, Pato Branco, Guarapuava e Paranaguá, com taxas que variaram de 1,10 a 2,44 casos por 1 mil nascidos vivos.

**Conclusão:**

O estudo apontou a heterogeneidade espacial dos casos de recém-nascidos com fissura orofacial no Paraná, tornando imprescindível a investigação das causas de fissura orofacial, o que contribui com a prevenção de novos casos e o fortalecimento de ações de saúde pública.

**Aspectos éticos**

A presente pesquisa respeitou os princípios éticos, obtendo os seguintes dados de aprovação: :

Comitê de ética em pesquisa: Universidade Estadual de Maringá

Número do parecer: 7.041.250

Data de aprovação: 29/8/2024

Certificado de apresentação de apreciação ética: 81990024.0.0000.0104

Registro do consentimento livre e esclarecido: Não se aplica.

## Introdução 

As fissuras orofaciais estão entre as anomalias craniofaciais mais recorrentes e vêm apresentando aumento no número de casos. Em 2020, a prevalência nacional de fissuras orofaciais foi 5,25 para cada 10 mil nascidos vivos. No país, entre 1999 e 2020, as regiões Sul e Sudeste possuíam índices mais elevados, sendo de 6,34 e 4,26 para cada 10 mil nascidos vivos (1). No Sul do Brasil, entre 2008 e 2017, identificou-se que a fissura orofacial em Santa Catarina foi 74,6 por 100 mil nascidos vivos, seguida do Rio Grande do Sul, com 71,8 por 100 mil nascidos vivos, e do Paraná, com 68,4 por 100 mil nascidos vivos (2).

Os casos variam de acordo com fatores raciais, geográficos e socioeconômicos (3). Entre 1999 e 2020 no Brasil, foi apontada a predominância de indivíduos do sexo masculino, com fissura de lábio e palato, de raça/cor da pele branca (1). Na Coreia do Sul, entre crianças com fissura labial e/ou de palato nascidos entre 2006 e 2018, identificou-se que crianças com fissura orofacial possuíam mais riscos de nascimento prematuro e morte neonatal, quando comparadas aos recém-nascidos sem anomalia (3).

Essa malformação pode acarretar complicações na amamentação e alimentação, alterações na fala, desafios psicossociais, problemas com a autoimagem e dificuldades com a resiliência. Muitos desses problemas podem afetar o sistema familiar como um todo, seja por desarranjos familiares, crises conjugais as quais culminam em divórcio, distanciamento dos outros filhos e abandono do emprego (4-6).

Apesar da relevância do tema, há escassez de estudos nacionais que explorem a distribuição espacial das fissuras orofaciais. Foram encontrados estudos internacionais que analisaram a distribuição espacial da malformação, realizados em Cuba, no Irã, na China e na Coreia do Sul, os quais permitiram identificar os locais com maior número de casos de fissura orofacial, contribuindo para a identificação da etiologia por fatores ambientais (7-10). No Brasil, no entanto, essa abordagem ainda é pouco explorada, limitando a compreensão da distribuição dos casos.

Este estudo mostrou-se necessário para contribuir no mapeamento epidemiológico das fissuras e teve como objetivo examinar a distribuição geográfica e a autocorrelação espacial dos casos de fissura orofacial em recém-nascidos no Paraná.

## Métodos 

### Delineamento do estudo

Tratou-se de estudo epidemiológico com as taxas de fissura orofacial em nascidos vivos no Paraná, entre 2018 e 2022, utilizando o Sistema de Informações sobre Nascidos Vivos. A realização do estudo atendeu às diretrizes do Strengthening the Reporting of OBservational studies in Epidemiology (STROBE) (11).

### Contexto 

Localizado na região Sul do Brasil, o Paraná abrange a área de 199.298 km^2^ e em 2022 sua população estimada era de 11.444.380 habitantes. O estado é dividido geograficamente em 10 mesorregiões e 399 municípios, com índice de desenvolvimento humano de 0,769, considerado como de elevado desenvolvimento humano (12,13). O estado é subdividido em 29 regiões imediatas, as quais correspondem a agrupamentos de municípios que possuem em comum um centro urbano como referência para sanar suas necessidades, principalmente nas áreas de saúde, educação, mão de obra, compras e consumo (14).

### Fonte de dados e mensuração

Os dados foram coletados em junho de 2024, utilizando o Sistema de Informações sobre Nascidos Vivos e as estimativas populacionais, que constam no Departamento de Informação e Informática do Sistema Único de Saúde (15,16).

Para o mapeamento da distribuição espacial dos nascidos vivos com fissura orofacial, foram calculadas as taxas para cada um dos municípios paranaenses, considerando a análise a ser realizada. As taxas foram calculadas utilizando a razão entre os nascidos vivos com fissura orofacial e a população de nascidos vivos em cada ano e local, multiplicada por 1 mil.

### Métodos estatísticos

Para a análise espacial, foi considerado o critério de vizinhança espacial e contiguidade do tipo Queen entre os municípios do Paraná. Realizou-se o cálculo do índice de Moran global e foi avaliado o grau de autocorrelação espacial do nascimento de recém-nascidos com fissura orofacial entre os municípios. 

O índice de Moran local foi aplicado para identificar aglomerados espaciais, e estes foram classificados em três tipos: alto-alto – altas taxas em municípios cercados por áreas igualmente elevadas; baixo-baixo – baixas taxas em municípios rodeados por áreas com baixas taxas; e baixo-alto – baixas taxas em cidades que fazem limites com áreas que possuem altas taxas (17).

Foram utilizados os softwares QGIS para distribuição espacial e GeoDa para identificar aglomerados espaciais. Para a análise exploratória dos dados e as modelagens, utilizaram-se o software R, versão 4.0.2 (R Core Team, 2020), e o R-INLA, versão 20.03.17. Para a elaboração da distribuição espacial, utilizou-se a base cartográfica do Paraná, contendo os limites municipais, disponível on-line em formato vetorial geográfico (18).

## Resultados 

Foram analisados 574 casos de recém-nascidos com fissura orofacial no Paraná, entre 2018 e 2022, distribuídos em 163 municípios do estado. O índice de Moran global indicou autocorrelação positiva forte (p-valor 0,001). As taxas suavizadas tiveram sua distribuição espacial apresentada na Figura 1A, em que os menores gradientes representaram menores taxas, e os gradientes mais pigmentados apresentaram maiores taxas, variando de 0,00 a 2,44 casos por 1 mil nascidos vivos. As regiões imediatas com taxas elevadas (entre 2,44) foram principalmente no sudoeste e no norte do estado: Paranaguá (cidade portuária), Pato Branco, Francisco Beltrão, Dois Vizinhos, Foz do Iguaçu, Telêmaco Borba e Cornélio Procópio/Bandeirantes. 

**Figura 1:** Distribuição espacial das taxas de fissuras orofaciais suavizadas a cada 1 mil nascidos vivos (A) e seus aglomerados (B). Paraná, 2018-2022

As regiões imediatas que apresentaram menores taxas suavizadas de recém-nascidos com fissura orofacial estavam localizadas no nordeste e noroeste do Paraná, sendo Loanda, Umuarama, Campo Mourão, Santo Antônio da Platina, Cornélio Procópio/Bandeirantes, Ibaiti, Irati e União da Vitória, variando de 0,00 a 0,37 casos por 1 mil nascidos vivos.

Os resultados da autocorrelação espacial local indicaram padrão de municípios com altas taxas cercados por municípios com taxas elevadas (alto-alto), significativo no sudoeste do estado, e municípios com baixas taxas cercados por municípios com baixas taxas (baixo-baixo), principalmente no nordeste e noroeste do estado (Figura 1B).

Os casos de fissura orofacial apresentaram aglomerados do tipo alto-alto nas regiões imediatas de Londrina, Cornélio Procópio/Bandeirantes, Ivaiporã, Cascavel, Foz do Iguaçu, Francisco Beltrão, Dois Vizinhos, Laranjeiras do Sul/Quedas do Iguaçu, Pato Branco, Guarapuava e Paranaguá.

As regiões imediatas de Loanda, Paranavaí, Paranacity, Cornélio Procópio/Bandeirantes, Santo Antônio da Platina, Ibaiti, Umuarama, Toledo, Cascavel, Campo Mourão, União da Vitória e Irati apresentaram aglomerados do tipo baixo-baixo.

## Discussão 

O padrão espacial dos casos de fissura orofacial no Paraná evidenciou distribuição não aleatória, com agrupamentos que apresentaram altas taxas principalmente no sudoeste e no norte do estado. Esse padrão indica que podem existir fatores comuns entre as regiões, que influenciam a ocorrência da malformação.

Por se tratar de estudo epidemiológico de distribuição espacial, houve algumas limitações na interpretação dos resultados. Não foi possível estabelecer relação causal ou identificar os fatores etiológicos para a ocorrência das fissuras orofaciais. A ausência de informações detalhadas não permitiu mensurar os fatores de risco conhecidos para as fissuras orofaciais em cada região.

Apesar das limitações, os padrões identificados sugeriram desigualdades estruturais entre os municípios. A disparidade de casos de fissura orofacial entre as cidades do Paraná pode estar relacionada ao desenvolvimento econômico das regiões e o acesso à saúde. Evidenciou-se que a maioria dos casos de recém-nascidos com fissuras orofaciais ocorreu em mães com menor nível de escolaridade, menor renda e acesso inadequado às consultas de pré-natal, o que indica a importância do contexto social para a saúde infantil (19). 

Destacou-se a relevância do consumo de ácido fólico antes e durante a gestação para evitar os casos de fissuras orofaciais (20,21). Uma metanálise conduzida por pesquisadores do Canadá confirmou, em seus achados, que o uso do ácido fólico é fator protetor para as fissuras orofaciais, principalmente quando utilizada antes da concepção (20). Na Etiópia, entre 2016 e 2018, 64,5% das mães que não utilizaram ácido fólico tiveram filhos com fissura orofacial (21). 

A adesão à suplementação pode estar relacionada à qualidade do pré-natal e ao conhecimento das gestantes (21). Avaliaram-se as características do pré-natal em relação às orientações oferecidas às gestantes de uma maternidade de Londrina. Nesse contexto, foi identificado que as mulheres receberam um número ideal de atendimentos, no entanto a qualidade da assistência variou de intermediária a inadequada, com acesso precário a informações no pré-natal (22). Identificou-se que a região de Londrina apresentou aglomerados do tipo alto-alto para os casos de fissura orofacial, que puderam estar relacionados a esses fatores.

Os fatores confundidores como multiparidade, dieta inadequada com nutrientes em níveis inferiores ao recomendado e idade materna avançada também devem ser considerados na investigação dos casos de fissuras orofaciais, o que evidencia a complexidade da malformação (23).

No sul do Irã, áreas urbanas concentravam mais casos de fissuras orofaciais do que em áreas rurais, sugerindo o risco de exposição materna a quantidades elevadas de poluentes do ar em grandes centros, devido ao grau de urbanização e à emissão de poluentes industriais (8). Tal fato pode ser aplicado aos centros urbanos do Paraná, como Londrina, centro reconhecido pelo comércio e pela indústria, que apresentou altas taxas de fissura orofacial. 

As causas das fissuras orofaciais, assim como outras malformações congênitas, não são completamente elucidadas. No entanto, nota-se a importância da interação entre os aspectos genéticos e ambientais. Entre os ambientais, a exposição a agrotóxicos aumenta consideravelmente as chances de desenvolvimento de fissura orofacial, associadas ou não a outras anomalias (24,25). Os agrotóxicos são muito utilizados no Paraná, visto que o estado é o segundo maior produtor de grãos (26) e ocupa a segunda posição nacional no uso de agrotóxicos. Entre 2013 e 2020, o consumo de agrotóxicos no estado aumentou 14,55%, com aumento de 8,87 kg/ha para 9,82 kg/ha. As regiões centro-sul, oeste e centro oriental apresentaram valores superiores à média estadual. Esse uso intensivo pode estar relacionado à maior incidência de fissuras orofaciais em regiões como Cascavel e Londrina, onde o consumo por hectare é elevado (27).

Entre lactantes residentes em Francisco Beltrão, encontrou-se a presença de glifosato (herbicida de amplo espectro muito utilizado no sudoeste do Paraná) em todas as amostras coletadas de leite materno, assim como na água, revelando que a população estava completamente exposta aos agrotóxicos. Os dados corroboram o achado deste estudo, pois o índice de fissura orofacial em recém-nascidos da região imediata de Francisco Beltrão esteve entre os mais altos (28).

Os aglomerados do tipo baixo-baixo em cidades localizadas principalmente no nordeste e no noroeste paranaenses podem não necessariamente refletir menor risco, mas podem estar relacionados às subnotificações dos casos de fissura orofacial em recém-nascidos decorrentes da rotatividade dos profissionais da saúde, da falta de capacitação, ou, ainda, do baixo investimento no rastreio, diagnóstico e acesso ao tratamento de crianças com essa anomalia congênita (19,29).

Este estudo contribuiu ao revelar parcialmente o panorama de casos de fissura orofacial no Paraná e foi inovador ao utilizar um método de análise pouco adotado nacionalmente para essa malformação. As descobertas sugeriram os locais de risco para recém-nascidos com fissura orofacial, o que fornecem aos gestores de saúde subsídios para refletir sobre o acesso à assistência pré-natal, bem como sobre os indicadores de risco em nível de área, os quais devem ser considerados prioridade ao desenvolver medidas para diminuir a ocorrência de fissura de lábio e/ou palato (9).

A pesquisa apontou a heterogeneidade espacial das taxas de nascimentos com fissura orofacial no Paraná. Identificaram-se agrupamentos municipais com taxas elevadas de fissura orofacial em recém-nascidos, indicando possíveis desigualdades regionais relacionadas a fatores sociais e ambientais e acesso à saúde. Embora o estudo não tenha feito inferências causais, os achados sugeriram que regiões com maior incidência podem refletir tanto maior exposição a riscos quanto melhor detecção, enquanto áreas com baixa incidência podem indicar subnotificação. 

Torna-se imprescindível a investigação das variáveis que possam estar interferindo nas taxas de fissura orofacial no Paraná, de modo a fortalecer as ações de saúde pública e prevenir o aumento dos casos. Além disso, deve-se investigar, de forma mais detalhada, os fatores ambientais, como a exposição a agrotóxicos e poluentes, que podem contribuir para o aumento da incidência nas regiões urbanas e agrícolas. 

Propõe-se a execução de outras pesquisas relacionadas ao tema, com delineamentos metodológicos variados e análises distintas. Esses estudos devem fornecer subsídios mais profundos que corroborem o desenvolvimento de estratégias preventivas e a adaptação das políticas públicas às realidades regionais.
